# Association between infrastructure and observed quality of care in 4 healthcare services: A cross-sectional study of 4,300 facilities in 8 countries

**DOI:** 10.1371/journal.pmed.1002464

**Published:** 2017-12-12

**Authors:** Hannah H. Leslie, Zeye Sun, Margaret E. Kruk

**Affiliations:** Department of Global Health and Population, Harvard T.H. Chan School of Public Health, Boston, Massachusetts, United States of America; London School of Hygiene and Tropical Medicine, UNITED KINGDOM

## Abstract

**Background:**

It is increasingly apparent that access to healthcare without adequate quality of care is insufficient to improve population health outcomes. We assess whether the most commonly measured attribute of health facilities in low- and middle-income countries (LMICs)—the structural inputs to care—predicts the clinical quality of care provided to patients.

**Methods and findings:**

Service Provision Assessments are nationally representative health facility surveys conducted by the Demographic and Health Survey Program with support from the US Agency for International Development. These surveys assess health system capacity in LMICs. We drew data from assessments conducted in 8 countries between 2007 and 2015: Haiti, Kenya, Malawi, Namibia, Rwanda, Senegal, Tanzania, and Uganda. The surveys included an audit of facility infrastructure and direct observation of family planning, antenatal care (ANC), sick-child care, and (in 2 countries) labor and delivery. To measure structural inputs, we constructed indices that measured World Health Organization-recommended amenities, equipment, and medications in each service. For clinical quality, we used data from direct observations of care to calculate providers’ adherence to evidence-based care guidelines. We assessed the correlation between these metrics and used spline models to test for the presence of a minimum input threshold associated with good clinical quality. Inclusion criteria were met by 32,531 observations of care in 4,354 facilities. Facilities demonstrated moderate levels of infrastructure, ranging from 0.63 of 1 in sick-child care to 0.75 of 1 for family planning on average. Adherence to evidence-based guidelines was low, with an average of 37% adherence in sick-child care, 46% in family planning, 60% in labor and delivery, and 61% in ANC. Correlation between infrastructure and evidence-based care was low (median 0.20, range from −0.03 for family planning in Senegal to 0.40 for ANC in Tanzania). Facilities with similar infrastructure scores delivered care of widely varying quality in each service. We did not detect a minimum level of infrastructure that was reliably associated with higher quality of care delivered in any service. These findings rely on cross-sectional data, preventing assessment of relationships between structural inputs and clinical quality over time; measurement error may attenuate the estimated associations.

**Conclusion:**

Inputs to care are poorly correlated with provision of evidence-based care in these 4 clinical services. Healthcare workers in well-equipped facilities often provided poor care and vice versa. While it is important to have strong infrastructure, it should not be used as a measure of quality. Insight into health system quality requires measurement of processes and outcomes of care.

## Abstract 2

In a cross-sectional study of 4,300 facilities in 8 countries, Hannah Leslie and colleagues examine the association between infrastructure and observed quality of care in 4 healthcare services.

## Author summary

**Why was this study done?:**

- Improved quality of care is increasingly recognized as a necessary step towards achievement of better population health outcomes in low- and middle-income countries.
- Much of the current measurement effort focuses on inputs to care.
- It is not known whether such measures provide insight on the quality of care delivered.

**What did the researchers do and find?:**

- We quantified facility infrastructure using international guidelines for readiness in each service applied to health facility audits in 8 countries; we defined quality of clinical care based on adherence to evidence-based protocols measured using direct observation in the same facility assessments.
- We calculated the level and correlation of infrastructure and average adherence to guidelines for each of 4 clinical services in this sample: family planning (1,842 facilities), antenatal care (1,407 facilities), delivery care (227 facilities), and sick-child care (4,038 facilities).
- Infrastructure scored higher than observed clinical quality in each service, and the correlation between the 2 was modest.

**What do these findings mean?:**

- Assessment of infrastructure is insufficient to estimate the quality of care delivered to women and children in need.
- Measurement priorities should be reassessed to support more timely information for quality improvement purposes and more pertinent information on the quality of care delivered for monitoring and comparison.

## Introduction

The first decade of the 2000s saw a dramatic increase in global health activity, with double-digit increases in international development assistance for health [1], reflecting the global focus on the HIV epidemic and intensified efforts to meet the Millennium Development Goals (MDGs) [2]. Two lessons learned in the pursuit of the health MDGs have particular salience for the current effort to achieve Sustainable Development Goal (SDG) 3: ensuring healthy lives and promoting well-being for all at all ages [3]. First, measurement can drive progress. With the assistance of several global initiatives, including the Countdown to 2015 and the Global Burden of Disease Study, countries closely tracked and compared population coverage of essential health services. As a result, remarkable global and national increases in coverage of services such as facility-based delivery and measles vaccination were achieved [2]. Improvements in health-related indicators that were MDG targets outstripped those in non-MDG targets by nearly 2-fold [4]. Second, for many conditions, increased access to care is insufficient to improve population health when care is of poor quality. In areas such as maternal and newborn health, studies from India, Malawi, and Rwanda have demonstrated that expanded access to formal healthcare has failed to yield survival benefits [5–7]. It is increasingly apparent that the path to achievement of SDG 3 will require similar attention to the measurement and improvement of healthcare quality as the MDG era brought to healthcare access [8,9].

Quality of care has been defined as the “degree to which health services for individuals and populations increase the likelihood of desired health outcomes and are consistent with current professional knowledge” [10]. Efforts to operationalize this broad definition have included the identification of key characteristics of quality, namely care that is safe, timely, effective, equitable, efficient, and people centered [11,12]. Health system theorists further agree that the delivery of high-quality care is contingent on adequate readiness of the health system or program and, once delivered, should yield impacts from improved health to client satisfaction [13,14]. In the same vein, measures of healthcare quality have traditionally been divided into 3 domains: structure or inputs to care, process or content of care, and outcomes of care [15]. Each domain has advantages and disadvantages: inputs are the necessary foundations for care but are not sufficient to describe its content or effects, process measures pertain directly to care delivery but are challenging to collect, and outcome measures assess the ultimate goal of the health system but reflect many factors beyond the health system itself.

In low- and middle-income countries (LMICs), information on healthcare quality is sparse [16]. A major source of data on health system performance has been standardized facility surveys, with over 100 unique surveys completed in the last 5 years alone [17–22]. Implementation of facility surveys is costly and typically supported by multilateral donor organizations such as the World Bank; World Health Organization (WHO); Global Fund for AIDS, Tuberculosis and Malaria; and the US Agency for International Development [20,21]. Among the most commonly used facility surveys is the Service Availability and Readiness Assessment (SARA), developed by WHO [22]. The SARA aims to measure facility readiness to provide essential care and hence focuses on inputs such as infrastructure, equipment, supplies, and health workers. Completion of a SARA survey costs a minimum of US$100,000 to generate national estimates for a small to medium country; more complex sampling to generate regional estimates can require several times that amount [23]. Other facility surveys also focus on input measures. For example, of 20 survey tools assessing health facility quality and readiness for family planning, 7 are limited to structural quality alone; across all 20 tools, indicators of structure are collected 5 times more frequently than indicators of process [18]. A review of 8,500 quality indicators used to assess performance-based financing programs found that over 90% measured structural aspects of quality [24]. The emphasis on input-based measures shapes health system research and monitoring: in the growing area of effective (quality-adjusted) coverage assessment, multiple studies look to input-based measures to estimate capacity to provide high-quality care [25–27].

The reliance on inputs to measure quality in LMICs reflects the notion that these are necessary for good care. However, while some inputs are clearly essential for care provision (e.g., health workers must be present; drugs must be in stock), it is not clear that overall availability of inputs is related to health processes or outcomes [28–31]. With growing attention to quality of care as a driver of future health gains and scarce resources available for measurement, selecting the right measures is important. Is infrastructure a reasonable proxy for quality of clinical care?

In this paper, we compare structural and process quality of 4 essential health services—family planning, antenatal care (ANC), delivery care, and sick-child care—using data from nationally representative samples of health facilities in 8 LMICs. The aims of this work are to describe facility inputs and observed adherence to guidelines for good clinical care for these services and to assess the strength of the relationship between these measures.

## Methods

### Ethical approval

The original survey implementers obtained ethical approval for data collection; primary data do not include identifiable patient information. The Harvard University Human Research Protection Program approved this secondary analysis as exempt from human subjects review.

### Study design and sample

The Service Provision Assessment (SPA) is a standardized survey designed to measure the capacity of health systems in LMICs. It is conducted by the Demographic and Health Survey Program of the US Agency for International Development in coordination with a national statistics agency in the countries surveyed. All health facilities in each country are listed, and a nationally representative sample is selected. The facility assessment includes a standard set of tools: an audit of facility services and resources, interviews with healthcare providers, and direct observation of the provision of clinical services.

In this analysis, we pooled data from all SPA surveys conducted between 2007 and 2015 that included observations of family planning, ANC, delivery care, and/or sick-child care. The surveys were from Haiti (2013), Kenya (2010), Malawi (2014), Namibia (2009), Rwanda (2007), Senegal (2013–2014), Tanzania (2015), and Uganda (2007). Surveys in Kenya, Senegal, Tanzania, and Uganda are nationally representative samples of the health system; those in Haiti, Malawi, Namibia, and Rwanda are censuses or near censuses. Observations were conducted in all services in all countries with the exception of delivery care, which was observed only in Kenya and Malawi. Patients are selected for observation using systematic random sampling from a list of those presenting for services on the day of the visit; assessment included up to 5 observations per provider and up to 15 observations per service. Children under 5 presenting with illness (as opposed to injury or skin or eye infection exclusively) were eligible for inclusion; when possible, new ANC clients and new family-planning clients were oversampled 2 to 1 relative to returning clients. For this analysis, we limited ANC observations to women’s first visit to the facility to standardize expected clinical actions. We excluded facilities with a single observation to limit variation.

### Facility infrastructure: Service readiness

We calculated infrastructure indices for each clinical service based on WHO definitions of service readiness [22]. We extracted the cross-cutting domains of basic amenities (e.g., safe water) and precautions for infection prevention (gloves, sanitizer) from the general service readiness index as an essential foundation for all services. We combined these with the 4 domains defined for each service-specific readiness score: staff and training, equipment, diagnostics (as applicable), and medicines and commodities. Each domain consists of specific tracer items such as functional blood pressure cuff, hemoglobin test, and valid iron pills for ANC (see S1 Table for items by service). Items that were not included on the survey for a given country were excluded from the calculation for that country. Some items were skipped if a facility did not have the service or capacity underlying the item—for example, stool microscopy in facilities without laboratory testing. We set these items to 0, reflecting the lack of capacity to use the item in that facility. In rare cases, facility managers provided invalid responses or no responses, leading to missing values; we imputed 0 for these items in the absence of evidence for their availability (and functional status) at the facility. Frequency of unasked, skipped, and missing items is reported in S1 Table. We computed domain scores as the mean availability of items and averaged across cross-cutting and service-specific domains to create an index from 0 to 1 for each service; each domain contributes equally to the final infrastructure index, regardless of the number of items it comprises.

### Observed clinical quality

Clinical observations consisted of an observer filling out a checklist of actions that providers are expected to complete during each patient visit; observers are members of the assessment team, typically nurses, who have completed training and evaluation on assessment procedures. We created indices of observed clinical quality for each service using international guidelines for evidence-based care or previously validated indices of quality [32–35]. Indices each contain between 16 and 22 items across domains such as patient history, physical exam, and counseling/management. S2 Table lists the items in each index and average performance by country. Each observation was scored based on percentage of items performed; observations were averaged within service in order to generate an average of quality of care per service delivered at each facility, weighted by the inverse probability of sampling clients within each facility.

### Analysis plan

We predefined infrastructure and observed clinical quality using international guidelines for both and identifying matching variables in the SPA. We identified unadjusted correlation as the appropriate analysis for a linear relationship and, in the absence of a predetermined threshold of inputs necessary for good clinical quality, used cross validation to rigorously test threshold options without overfitting to the observed data. We considered assessment of the full sample and of the sample limited to facilities with more than 1 observation; we selected the latter as the main analysis due to a priori concerns about measurement error in data from a single observation, i.e., that single observations may be less reliable than multiple observations in conveying underlying quality.

### Statistical analysis

We provided descriptive statistics of service-specific facility characteristics, including whether the facility is a hospital versus a health center, clinic, or dispensary; whether it was publicly or privately managed; whether it is located in an urban or rural area; and the number of observations per facility. We also calculated mean and standard deviation of the service-specific infrastructure index and observed clinical quality in each country and assessed correlation of infrastructure across services and clinical quality across services. We calculated the intraclass correlation (ICC) by country to quantify variation.

We generated scatterplots of infrastructure and observed clinical quality with a linear curve to visualize the association and calculated Pearson’s correlation coefficient for each association. The smooth curve in each scatterplot was fitted using a generalized additive model to capture potential nonlinear effect; the shaded area represents the 95% confidence interval around the smoothed curve. Histograms of infrastructure and observed clinical quality were plotted along the 2 axes. We divided facilities into quintiles of infrastructure and plotted median clinical quality and the interquartile range (IQR) across quintiles to visualize variability of process quality within levels of structural inputs.

We attempted to identify a minimum threshold of inputs required for good quality clinical care. We tested for nonlinearity in the relationship between infrastructure and observed quality by fitting linear spline models with a single knot. Because we do not have prior knowledge about the location of the knot, we started from a range of cutoff values between the minimum and maximum of infrastructure. For each cutoff value, we fit a linear spline model of observed clinical quality on infrastructure with a new variable taking the values of the marginal increase of service infrastructure above the cutoff value. We obtained prediction error using 10-fold cross validation for each candidate value [36]. We picked the cutoff value with the smallest prediction error as the location of the knot for the final model for each country and service. We assessed the statistical significance (*p* ≤ 0.05) of the marginal spline in this model to determine whether the spline meaningfully changed the association from the basic linear model.

Cross-country analyses are weighted so that each country contributes equally to the sample; within-country analyses are unweighted due to the restrictive selection criteria applied to the final analytic sample. Analyses were conducted in Stata version 14.1 (StataCorp, College Station, Texas) and R version 3.3.1 (the R Foundation for Statistical Computing).

## Results

Of 8,501 facilities selected, 8,254 (97.1%) were assessed; 4,354 facilities had at least one valid observation in the selected services (32,531 total observations). The analytic sample comprised 1,407 facilities for ANC, 1,842 for family planning, 227 for delivery, and 4,038 for sick-child care. Because observations were sampled based on availability of patients on the day of visit, facilities excluded from the analysis were disproportionately smaller clinics and health centers. Hospitals made up approximately 25% of the sample for ANC, family planning, and sick-child care and 71% of the facilities for delivery care (Table 1). Approximately 27% of facilities were privately managed, ranging from 22% in family planning to 30% in sick-child care. The number of observations per facility varied from 3.42 in ANC to 4.71 in sick-child care.

**Table 1:** Characteristics of facilities providing family planning, antenatal, sick-child, and delivery care in 8 countries, 2007–2015.

	Facilities with direct observation of the following:			
	Family planning (*N* = 1,842)	Antenatal care (*N* = 1,407)	Delivery care (*N* = 227)	Sick-child care (*N* = 4027)
**Facility Characteristics**				
Hospital^‡^	417 (23%)	413 (29%)	161 (71%)	798 (20%)
Private^§^	401 (22%)	383 (27%)	63 (28%)	1,227 (30%)
Urban^¶^	474 (39%)	364 (37%)	34 (33%)	915 (34%)
Observations per facility (mean, SD)	4.25 (2.00)	3.42 (1.68)	4.12 (2.98)	4.71 (2.09)
**Facilities in**				
Haiti	302 (16%)	221 (16%)	0 (0%)	515 (13%)
Kenya	228 (12%)	157 (11%)	124 (55%)	450 (11%)
Malawi	320 (17%)	194 (14%)	103 (45%)	679 (17%)
Namibia	187 (10%)	81 (6%)	0 (0%)	277 (7%)
Rwanda	155 (8%)	81 (6%)	0 (0%)	373 (9%)
Senegal	225 (12%)	121 (9%)	0 (0%)	561 (14%)
Tanzania	372 (20%)	447 (32%)	0 (0%)	908 (23%)
Uganda	53 (3%)	105 (7%)	0 (0%)	264 (7%)

Source, authors’ analysis of Service Provision Assessment data from 8 countries.

^‡^Facility is a hospital.

^§^Facility is managed by a private (nongovernmental or faith-based) authority.

^¶^Facility is in urban area. Note that only facilities in Haiti, Malawi, Senegal, and Tanzania have information on urban versus rural location.

**Abbreviation**: SD, standard deviation.

Facilities in the sample demonstrated moderate levels of infrastructure across all services (Table 2). Infrastructure was highest in family planning (averaging 0.70 in Rwanda to 0.80 in Kenya) and lowest in sick-child care (averaging 0.59 in Haiti and Rwanda to 0.70 in Namibia). Observed clinical quality was low in all services, with an average of 60% of clinical actions completed in ANC and delivery care compared to 48% in family planning and only 37% in sick-child care. Although infrastructure in different clinical areas was correlated by definition due to shared basic amenities and infection control domains, the magnitude of the correlation ranged from a minimum of 0.41 for delivery care and family planning to a maximum of 0.69 for ANC and sick-child care (Panel A in S3 Table). Correlation was lower for clinical quality across services, with negative correlation for delivery care with ANC and sick-child care and the largest correlation at 0.32 for ANC and family planning (Panel B in S3 Table). In all services, the ICC for within- versus between-country variance was higher for observed clinical quality (Panel C in S3 Table), indicating that clinical quality varied relatively more between countries than did infrastructure.

**Table 2:** Summary statistics of infrastructure and observed clinical quality in facilities providing family planning, antenatal, sick-child, and delivery care.

	Family planning (*N* = 1,842)		Antenatal care (*N* = 1,407)		Delivery care (*N* = 227)		Sick-child care (*N* = 4,027)	
	Infrastructure mean (SD)	Clinical quality mean (SD)	Infrastructure mean (SD)	Clinical quality mean (SD)	Infrastructure mean (SD)	Clinical quality mean (SD)	Infrastructure mean (SD)	Clinical quality mean (SD)
Haiti	0.76 (0.12)	0.36 (0.11)	0.65 (0.13)	0.47 (0.13)			0.59 (0.13)	0.30 (0.09)
Kenya	0.80 (0.13)	0.50 (0.16)	0.77 (0.12)	0.71 (0.14)	0.69 (0.10)	0.58 (0.16)	0.65 (0.14)	0.42 (0.15)
Malawi	0.72 (0.15)	0.39 (0.13)	0.67 (0.16)	0.48 (0.11)	0.71 (0.13)	0.62 (0.12)	0.62 (0.13)	0.31 (0.11)
Namibia	0.73 (0.09)	0.46 (0.12)	0.66 (0.09)	0.76 (0.10)			0.70 (0.09)	0.54 (0.14)
Rwanda	0.70 (0.11)	0.62 (0.23)	0.64 (0.10)	0.62 (0.15)			0.59 (0.12)	0.31 (0.13)
Senegal	0.76 (0.12)	0.45 (0.13)	0.75 (0.11)	0.63 (0.12)			0.67 (0.12)	0.30 (0.10)
Tanzania	0.76 (0.15)	0.49 (0.16)	0.70 (0.17)	0.60 (0.14)			0.62 (0.16)	0.32 (0.13)
Uganda	0.74 (0.12)	0.54 (0.21)	0.69 (0.15)	0.61 (0.14)			0.61 (0.14)	0.49 (0.17)

Source, authors’ analysis of Service Provision Assessment data from 8 countries.

Note that delivery care was not directly observed in countries other than Kenya and Malawi.

**Abbreviation**: SD, standard deviation.

**Fig 1:**
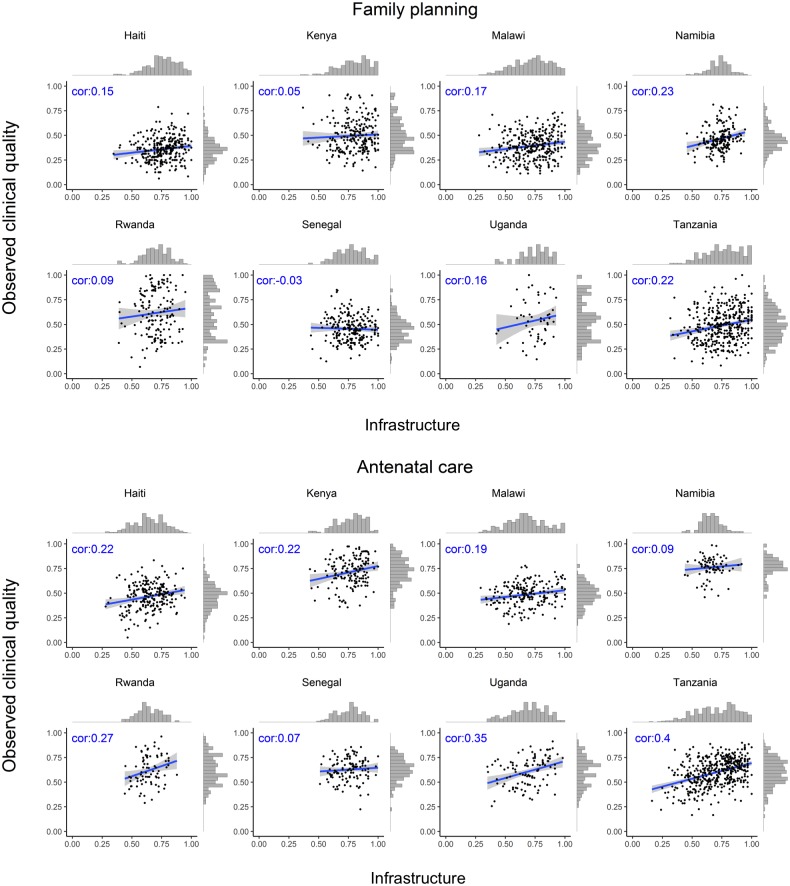
Association between infrastructure and observed clinical quality for family planning and antenatal care. cor, correlation coefficient.

**Fig 2:**
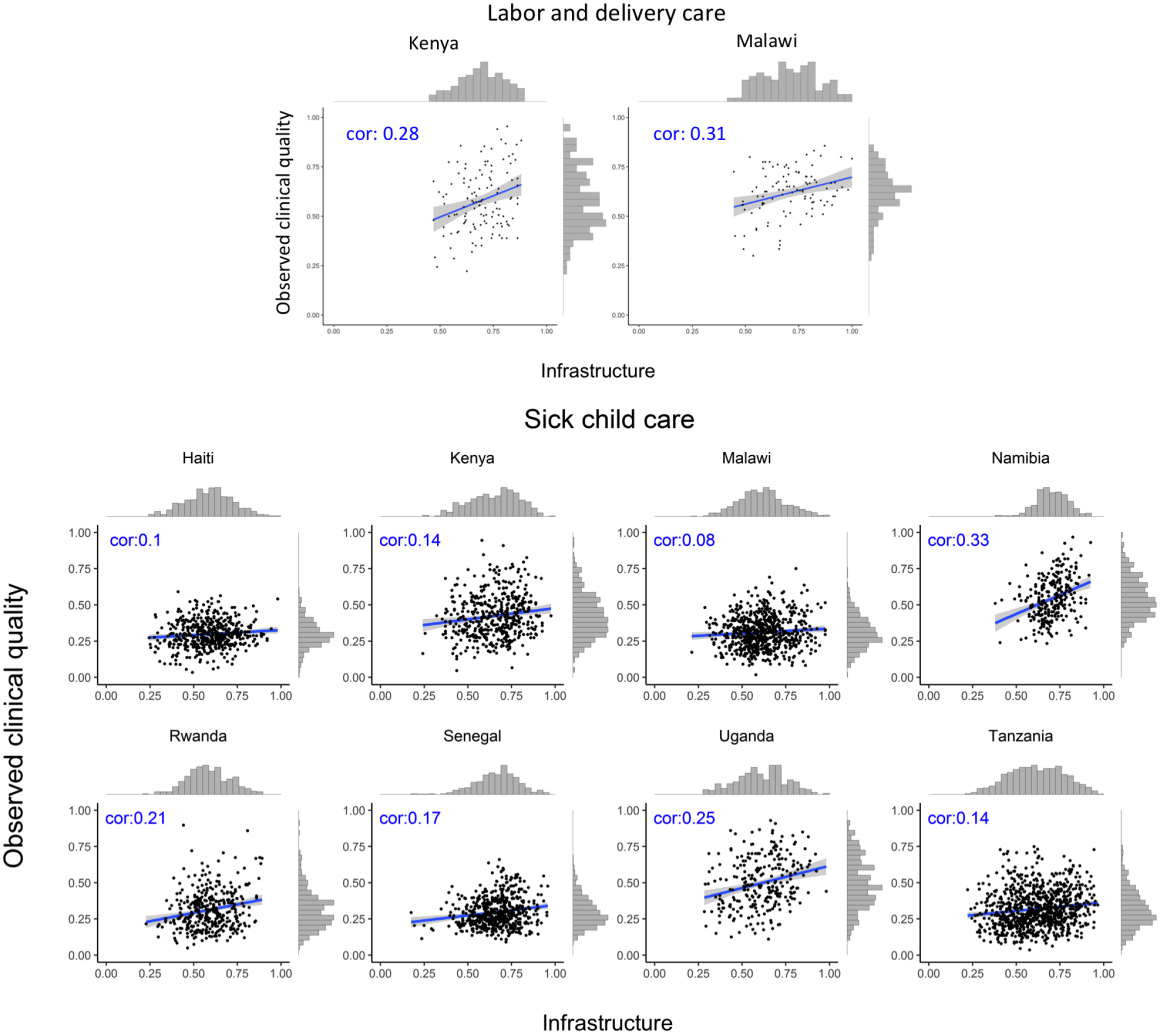
Association between infrastructure and observed clinical quality for labor and delivery care and sick-child care. cor, correlation coefficient.

The association between infrastructure and observed clinical quality for each service is shown by country in Fig 1 (family planning and ANC) and Fig 2 (labor and delivery care and sick-child care). The variation in observed clinical quality in particular is evident in the range of the scatter along the y-axis and the flatter histograms in most, though not all, plots. Across different countries and services, the association was consistently positive but weak, with highly variable magnitude across countries by service, ranging across all analyses from −0.03 (family planning in Senegal) to 0.40 (ANC in Tanzania). Median correlation across all services and countries was 0.20.

Fitting spline models failed to identify a significant inflection point in any country for any service (S1 Fig). The association between infrastructure and observed clinical quality was generally linear across countries and services.

**Fig 3:**
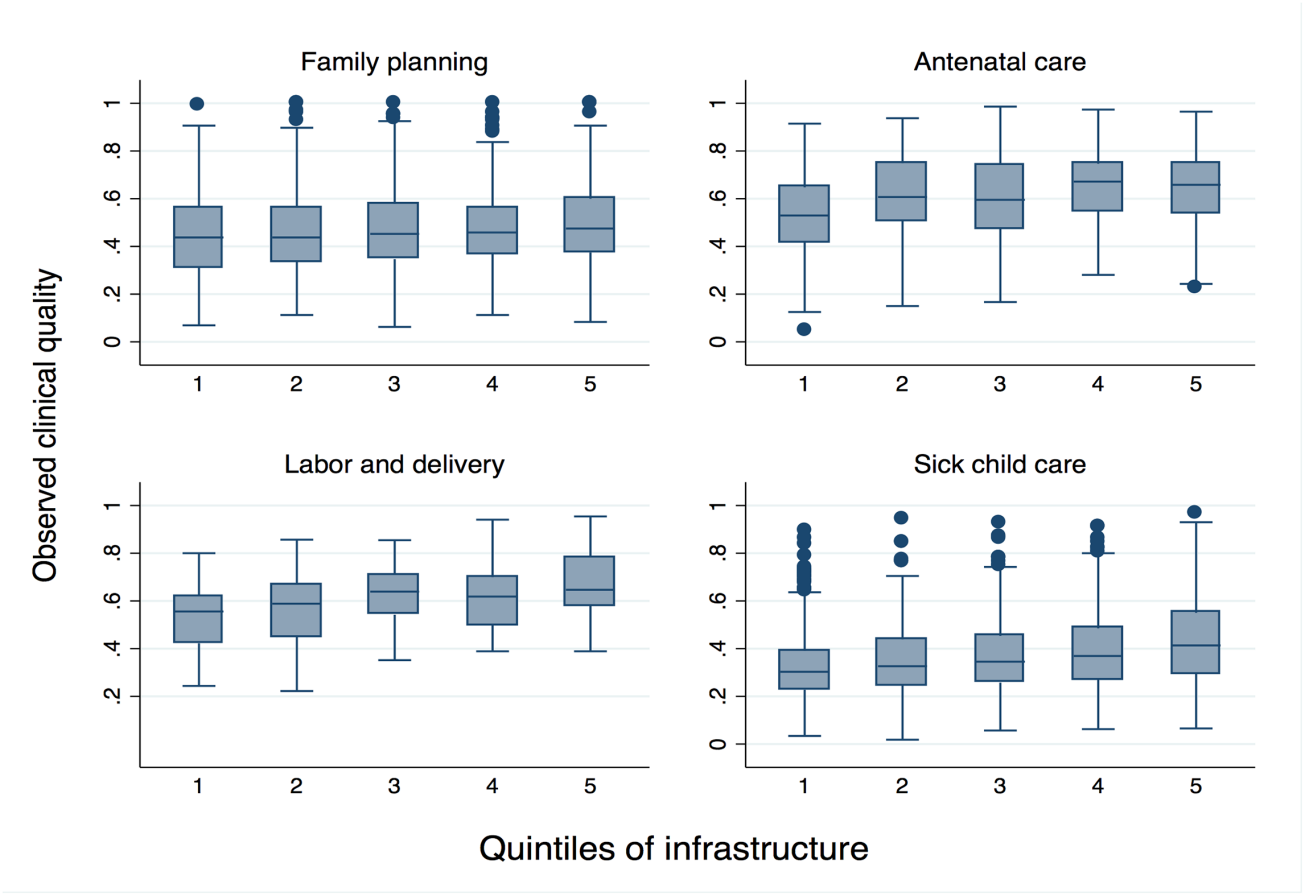
Range of observed clinical quality across quintiles of infrastructure.

The boxes in Fig 3 display median and IQR of observed quality by quintile of infrastructure pooled across all countries. The modest association between inputs and observed clinical quality are evident in ANC and delivery care in particular. Even as infrastructure increases, however, variability in observed clinical quality remains high: IQR in the highest quintile of infrastructure barely differs from that in the lowest quintile, with the exception of sick-child care, for which the IQR increases from 0.16 to 0.26 as infrastructure increases (Table 3).

**Table 3:** Median and interquartile range of observed clinical quality by quintile of infrastructure.

	Family planning			Antenatal care			Labor and delivery			Sick-child care		
Quintile of facility infrastructure	Infrastructure range	Median observed clinical quality	IQR observed clinical quality	Infrastructure range	Median observed clinical quality	IQR observed clinical quality	Infrastructure range	Median observed clinical quality	IQR observed clinical quality	Infrastructure range	Median observed clinical quality	IQR observed clinical quality
1	0.28–0.65	0.44	0.25	0.16–0.58	0.53	0.23	0.44–0.59	0.56	0.20	0.18–0.51	0.30	0.16
2	0.65–0.72	0.44	0.22	0.58–0.65	0.61	0.24	0.59–0.67	0.59	0.22	0.52–0.60	0.33	0.19
3	0.72–0.79	0.45	0.23	0.65–0.73	0.60	0.27	0.67–0.74	0.64	0.17	0.60–0.67	0.35	0.20
4	0.79–0.86	0.46	0.19	0.73–0.81	0.67	0.20	0.74–0.81	0.62	0.20	0.67–0.75	0.37	0.21
5	0.87–1.00	0.47	0.23	0.82–1.00	0.66	0.21	0.82–1.00	0.65	0.21	0.75–0.98	0.41	0.26

**Abbreviation**: IQR, interquartile range.

## Discussion

Across multiple clinical services in 8 countries, correlation between inputs and adherence to evidence-based care guidelines was weak: within each service, facilities with similar levels of infrastructure provided widely varying care. Observed clinical quality tended to be more variable and lower than infrastructure in nearly all countries and services, suggesting that using inputs as a proxy for quality of care as delivered would be both unreliable and systematically biased to overstate quality. These results were based on a sample stripped of likely outliers (facilities with a single observation of clinical care per service) in order to minimize noise in the association of inputs and process quality. Even in these generally larger facilities, gaps in readiness to provide essential care and particularly in observed clinical quality were evident in all services and countries. Although inputs to care should serve as an essential foundation for high-quality care, these data did not suggest the existence of a minimum threshold of inputs necessary for providing better care within the range of infrastructure observed here. It is possible that such a threshold exists at extremely low levels of facility infrastructure.

The deficiencies in facility infrastructure found in this study are similar to prior assessments of structural inputs [16,37,38] and suggest that even the hospitals and larger facilities overrepresented in this analysis lack key elements of basic amenities, equipment, and medications required to provide basic services. Cross-national estimates of process quality measures are scarcer, but a growing body of evidence from national and subnational studies attests to high variability and low attainment in measures of clinical process quality in low-resource settings [39–41]. Our finding of lower process quality than inputs affirm findings in diverse settings such as India [42], Bangladesh [43], and South Sudan [44].

Measuring the necessary inputs to care provided limited insight on the process quality of care delivered in primary care services as well as in more resource-intensive delivery care. These findings amplify a study of pay-for-performance interventions in Rwanda, demonstrating that increased availability of inputs for delivery care explained an insignificant fraction of increased delivery volume [45]. Although we would not expect perfect correlation due to the breadth of the infrastructure measures relative to the specific items of evidence-based care, the limited associations and high variability in observed clinical quality at similar levels of facility infrastructure was striking, even for well-equipped facilities. More surprisingly, some facilities were able to provide above-average care quality at quite low levels of infrastructure. While several of the elements of observed clinical quality in these services—particularly the primary care services of family planning, ANC, and sick-child care—could be completed with no supplies, rudimentary equipment such as thermometers and blood pressure cuffs are required. Low to modest correlation in the assessed measures suggests that performance on global standards for readiness bears little relevance for performance on global standards for provision of care. Our findings underscore the importance of direct measurement of the process of care as delivered to provide meaningful insight on the current state of quality and the key areas for improvement. The importance of measuring care processes is bolstered by the growing evidence of a know–do gap, in which providers often underperform their knowledge tests [40,41,46].

This work calls into question the utility of health facility assessments such as the SARA and other surveys focusing on inputs in their current configuration: if facility infrastructure is only weakly correlated with the delivery of care, it is likely to be even less correlated to outcomes. Subnational and cross-national comparison of inputs to care will thus serve little purpose in understanding how the health system is performing in improving population health. Assessment of infrastructure, including the functioning of basic amenities and equipment and the availability of medicines and supplies, is important for proper health system management, but such information is required on a local level and with high frequency if it is to be actionable. Procurement and other supply chain information systems offer a better source for this information than expensive and infrequent facility surveys. Bolstering their capacity—and in particular the analysis and use of such data for monitoring and improvement purposes—is a global health priority [47].

Given the limited resources for health system measurement—including health worker time—information collected must be justified based on its value in understanding and improving health system performance; methods of data collection should be optimized for the intended purpose. Health facility assessments can provide valuable standardized information on the health system just as Demographic and Health Surveys do on the population. Improvements to current health facility assessments should be pursued, including attempts to identify a minimum set of input indicators that reflect overall structural capacity and in standardizing indicators of healthcare processes or impacts that best capture the quality of care for subnational, national, and cross-national monitoring and performance assessment. Consideration of a range of quality indicators and methods to collect them is warranted, such as vital registration, focused direct observations, patient exit and community surveys, and stronger measures of healthcare-sensitive health outcomes, including patient-reported outcomes, in facilities and after discharge.

To our knowledge, this is the first multi-country, multiservice comparison of inputs and process quality measures in low-resource settings. The analysis was based on 32,566 direct observations of clinical care from the most comprehensive health facility assessment in widespread use [17]. We limited our sample to facilities with multiple observations to minimize the impact of single, potentially nonrepresentative observations and defined infrastructure using the essential equipment and supplies pertinent to the type of care being observed, as defined by WHO. However, there are several limitations to this study. Direct observation of care can increase provider efforts via the Hawthorne effect [48], although limiting to facilities with multiple observations should mitigate its impact on the results. Observer error or inability to observe procedures taking place prior to the clinical encounter could introduce variability in measurement unrelated to the quality of clinical care provided. Variation in assessing each visit could attenuate the relationship between infrastructure and average quality [49]. The small number of countries precludes assessment of changes in the observed associations over time; the selected surveys spanned 2007–2015; while it is likely that efforts to achieve the MDGs affected facility infrastructure and observed clinical quality in these countries, it is not possible to assess such effects in these data or to determine whether such changes might have strengthened or weakened the association between them. The cross-sectional nature of the data makes it impossible to identify associations between long-term availability of equipment and supplies and clinical care quality as well as to disentangle reverse causality such as shortages due to high patient demand. These factors could contribute to the variability in the observed data; any data source addressing these concerns would require longitudinal data collection.

As the quality of care assumes a more prominent role in national and global efforts to improve population health outcomes, accurate measurement is vital. Healthcare providers and physical inputs, such as buildings, medicines, and equipment, are an essential foundation for delivering healthcare. However, we found that these structural measures provide little insight on the quality of services delivered to patients. Expanding measurement of processes and outcomes of care is imperative to achieve better health outcomes and improve performance of health systems.

## Supporting information

S1 STROBE Checklist (DOC)

S1 Table Summary statistics of items composing country-specific infrastructure. (DOCX)

S2 Table Summary statistics of items composing country-specific clinical quality. (DOCX)

S3 Table Correlation of infrastructure and observed clinical quality by service. (DOCX)

S1 Fig Assessment of potential nonlinearity in associations between infrastructure and clinical quality in 4 health services. (DOCX)

## Abbreviations

ANC: antenatal care
ICC: intraclass correlation
IQR: interquartile range
LMICs: low- and middle-income countries
MDG: Millennium Development Goal
SARA: Service Availability and Readiness Assessment
SDG: Sustainable Development Goal
SPA: Service Provision Assessment
WHO: World Health Organization
