# Supplementary material for: Association between infrastructure and observed quality of care in 4 healthcare services: A cross-sectional study of 4,300 facilities in 8 countries
Source: PLoS Med. 2017 Dec 12;14(12):e1002464. doi: 10.1371/journal.pmed.1002464 (PMC5726617; doi:10.1371/journal.pmed.1002464)
Supplement: S1 Table — (DOCX) [file pmed.1002464.s002.docx]

**S1 Table**: Summary statistics of items composing country-specific infrastructure measures

|  | Total sample (N) | | | | Frequency by country (mean) | | | | | | | |
| --- | --- | --- | --- | --- | --- | --- | --- | --- | --- | --- | --- | --- |
| Items | Valid | Skipped by country | Skipped by facility | Missing | Haiti | Kenya | Malawi | Namibia | Rwanda | Senegal | Tanzania | Uganda |
| Service: all (N=4354) |  |  |  |  |  |  |  |  |  |  |  |  |
| Domain: basic amenities |  |  |  |  |  |  |  |  |  |  |  |  |
| Electricity | 4354 | 0 | 0 | 0 | 0.57 | 0.45 | 0.32 | 0.48 | 0.51 | 0.37 | 0.46 | 0.51 |
| Water | 4343 | 0 | 0 | 11 | 0.42 | 0.50 | 0.59 | 0.57 | 0.35 | 0.67 | 0.43 | 0.50 |
| Privacy | 4354 | 0 | 0 | 0 | 0.98 | 1.00 | 0.99 | 1.00 | 1.00 | 1.00 | 0.99 | 0.99 |
| Toilet | 4028 | 292 | 0 | 34 | 0.48 | 0.99 | 0.31 | NA | 0.94 | 0.90 | 0.54 | 0.94 |
| Communication | 4351 | 0 | 0 | 3 | 0.69 | 0.61 | 0.75 | 0.87 | 0.68 | 0.57 | 0.44 | 0.55 |
| Computer & internet | 4354 | 0 | 0 | 0 | 0.35 | 0.38 | 0.22 | 0.12 | 0.13 | 0.37 | 0.28 | 0.26 |
| Ambulance | 4354 | 0 | 0 | 0 | 0.17 | 0.40 | 0.30 | 0.19 | 0.24 | 0.39 | 0.32 | 0.49 |
| Domain: infection prevention |  |  |  |  |  |  |  |  |  |  |  |  |
| Final sharps disposal, e.g. incineration | 4255 | 0 | 2 | 97 | 0.66 | 0.74 | 0.81 | 0.89 | 0.61 | 0.90 | 0.62 | 0.29 |
| Final infectious waste disposal, e.g. incineration | 4300 | 0 | 8 | 46 | 0.58 | 0.64 | 0.77 | 0.64 | 0.56 | 0.58 | 0.62 | 0.28 |
| Sharps disposal box | 4354 | 0 | 0 | 0 | 0.97 | 1.00 | 0.99 | 1.00 | 1.00 | 0.99 | 0.99 | 0.97 |
| Waste bin | 4354 | 0 | 0 | 0 | 0.39 | 0.54 | 0.69 | 0.63 | 0.99 | 0.83 | 0.80 | 0.84 |
| Surface disinfectant | 4354 | 0 | 0 | 0 | 0.89 | 0.95 | 0.90 | 0.93 | 0.15 | 0.98 | 0.92 | 0.76 |
| Syringe | 4354 | 0 | 0 | 0 | 0.97 | 1.00 | 0.99 | 0.99 | 1.00 | 0.90 | 0.97 | 0.98 |
| Soap and water or hand sanitizer | 4354 | 0 | 0 | 0 | 0.90 | 0.94 | 0.88 | 1.00 | 0.94 | 0.98 | 0.92 | 0.94 |
| Gloves | 4354 | 0 | 0 | 0 | 0.97 | 1.00 | 0.99 | 1.00 | 1.00 | 0.99 | 0.97 | 1.00 |
| Infection prevention guidelines | 4354 | 0 | 0 | 0 | 0.42 | 0.36 | 0.74 | 0.20 | 0.08 | 0.76 | 0.60 | 0.24 |
| Service: family planning (N=1842) |  |  |  |  |  |  |  |  |  |  |  |  |
| Domain: Staff and training |  |  |  |  |  |  |  |  |  |  |  |  |
| Guidelines for family planning | 1842 | 0 | 0 | 0 | 0.70 | 0.64 | 0.64 | 0.51 | 0.32 | 0.72 | 0.85 | 0.55 |
| Training in family planning | 1836 | 0 | 0 | 6 | 0.64 | 0.60 | 0.58 | 0.24 | 0.50 | 0.70 | 0.60 | 0.38 |
| Domain: equipment |  |  |  |  |  |  |  |  |  |  |  |  |
| Blood pressure cuff | 1842 | 0 | 0 | 0 | 0.90 | 0.93 | 0.70 | 0.94 | 0.94 | 0.98 | 0.76 | 0.87 |
| Domain: medicine and commodities |  |  |  |  |  |  |  |  |  |  |  |  |
| Oral contraceptive | 1834 | 0 | 5 | 3 | 0.94 | 0.99 | 0.96 | 0.99 | 0.97 | 0.62 | 0.93 | 1.00 |
| Injection contraceptive | 1837 | 0 | 4 | 1 | 0.99 | 0.97 | 0.99 | 1.00 | 0.99 | 0.66 | 0.96 | 0.98 |
| Male condom | 1834 | 0 | 8 | 0 | 0.94 | 0.95 | 0.72 | 0.98 | 0.85 | 0.77 | 0.83 | 0.91 |
| Service: Antenatal care (N=1407) |  |  |  |  |  |  |  |  |  |  |  |  |
| Domain: Staff and training |  |  |  |  |  |  |  |  |  |  |  |  |
| Guidelines for antenatal care | 1378 | 0 | 0 | 29 | 0.38 | 0.76 | 0.68 | 0.15 | 0.31 | 0.63 | 0.65 | 0.39 |
| Training in ANC | 1401 | 0 | 0 | 6 | 0.81 | 0.78 | 0.90 | 0.43 | 0.43 | 0.83 | 0.88 | 0.39 |
| Domain: equipment |  |  |  |  |  |  |  |  |  |  |  |  |
| Blood pressure cuff | 1407 | 0 | 0 | 0 | 0.94 | 0.92 | 0.71 | 0.95 | 0.95 | 0.93 | 0.86 | 0.79 |
| Domain: diagnostics |  |  |  |  |  |  |  |  |  |  |  |  |
| Hemoglobin test | 1219 | 0 | 187 | 1 | 0.05 | 0.27 | 0.33 | 0.10 | 0.15 | 0.06 | 0.50 | 0.64 |
| Urine dipstick test | 1220 | 0 | 187 | 0 | 0.48 | 0.79 | 0.20 | 0.99 | 0.67 | 0.67 | 0.58 | 0.75 |
| Domain: medicine and commodities |  |  |  |  |  |  |  |  |  |  |  |  |
| Iron tablet | 1405 | 0 | 0 | 2 | 0.86 | 0.80 | 0.97 | 0.94 | 0.86 | 0.97 | 0.95 | 0.64 |
| Folate tablet | 1405 | 0 | 0 | 2 | 0.86 | 0.89 | 0.97 | 0.89 | 0.79 | 0.93 | 0.96 | 0.75 |
| Tetanus toxoid vaccine | 1396 | 0 | 0 | 11 | 0.37 | 0.96 | 0.69 | 0.96 | 0.90 | 0.93 | 0.92 | 0.92 |
| IPT tablet | 1184 | 221 | 2 | 0 | NA | 0.96 | 0.98 | 0.70 | 0.99 | 0.91 | 0.53 | 0.99 |
| Insecticide treated nets | 960 | 447 | 0 | 0 | NA | 0.82 | 0.80 | 0.68 | 0.96 | NA | 0.11 | NA |
| Service: sick child care (N=4027) |  |  |  |  |  |  |  |  |  |  |  |  |
| Domain: staff and training |  |  |  |  |  |  |  |  |  |  |  |  |
| Guidelines for sick-child care | 4019 | 0 | 0 | 8 | 0.34 | 0.50 | 0.41 | 0.44 | 0.19 | 0.49 | 0.63 | 0.37 |
| Guidelines for child growth monitoring | 2317 | 1364 | 346 | 0 | 0.32 | NA | 0.33 | NA | NA | 0.61 | 0.30 | NA |
| Training in sick-child care | 4003 | 0 | 0 | 24 | 0.38 | 0.34 | 0.31 | 0.21 | 0.18 | 0.37 | 0.29 | 0.35 |
| Domain: equipment |  |  |  |  |  |  |  |  |  |  |  |  |
| Pediatric scale | 4025 | 0 | 0 | 2 | 0.86 | 0.94 | 0.84 | 0.94 | 0.74 | 0.91 | 0.65 | 0.85 |
| Height board | 2767 | 914 | 346 | 0 | 0.57 | 0.53 | 0.76 | NA | NA | 0.74 | 0.37 | NA |
| Thermometer | 4025 | 0 | 0 | 2 | 0.97 | 0.91 | 0.87 | 0.97 | 0.90 | 0.98 | 0.84 | 0.79 |
| Stethoscope | 2663 | 1364 | 0 | 0 | 0.98 | NA | 0.93 | NA | NA | 0.99 | 0.93 | NA |
| Growth chart | 2317 | 1364 | 346 | 0 | 0.48 | NA | 0.54 | NA | NA | 0.72 | 0.67 | NA |
| Domain: diagnostics |  |  |  |  |  |  |  |  |  |  |  |  |
| Hemoglobin test | 3273 | 0 | 751 | 3 | 0.06 | 0.27 | 0.23 | 0.07 | 0.19 | 0.07 | 0.46 | 0.45 |
| Stool microscopy | 3272 | 0 | 751 | 4 | 0.44 | 0.75 | 0.23 | 0.94 | 0.41 | 0.29 | 0.43 | 0.60 |
| Malaria test | 3274 | 0 | 751 | 2 | 0.62 | 0.32 | 0.78 | 0.97 | 0.83 | 0.71 | 0.67 | 0.20 |
| Domain: medicine and commodities |  |  |  |  |  |  |  |  |  |  |  |  |
| Oral rehydration salts | 3980 | 0 | 46 | 1 | 0.71 | 0.95 | 0.94 | 0.97 | 0.92 | 0.85 | 0.88 | 0.91 |
| Amoxicillin | 3980 | 0 | 46 | 1 | 0.93 | 0.79 | 0.92 | 0.96 | 0.85 | 0.80 | 0.88 | 0.44 |
| Co-trimoxazole suspension | 3361 | 637 | 29 | 0 | 0.72 | 0.60 | 0.74 | 0.89 | NA | 0.79 | 0.78 | NA |
| Paracetamol syrup | 3361 | 637 | 29 | 0 | 0.78 | 0.90 | 0.72 | 0.93 | NA | 0.81 | 0.67 | NA |
| Vitamin A | 3981 | 0 | 46 | 0 | 0.56 | 0.87 | 0.47 | 0.95 | 0.35 | 0.56 | 0.66 | 0.73 |
| Deworming tablet | 3981 | 0 | 46 | 0 | 0.92 | 0.94 | 0.96 | 0.88 | 0.91 | 0.88 | 0.90 | 0.92 |
| Zinc | 2638 | 1364 | 25 | 0 | 0.56 | NA | 0.83 | NA | NA | 0.70 | 0.53 | NA |
| Service: delivery |  |  |  |  |  |  |  |  |  |  |  |  |
| Domain: staff and training |  |  |  |  |  |  |  |  |  |  |  |  |
| Delivery guidelines | 227 | 0 | 0 | 0 |  | 0.48 | 0.48 |  |  |  |  |  |
| Training in IMPAC | 227 | 0 | 0 | 0 |  | 0.00 | 0.38 |  |  |  |  |  |
| Domain: equipment |  |  |  |  |  |  |  |  |  |  |  |  |
| Emergency transport | 227 | 0 | 0 | 0 |  | 0.81 | 0.55 |  |  |  |  |  |
| Sterilization equipment | 220 | 0 | 0 | 7 |  | 0.94 | 0.56 |  |  |  |  |  |
| Exam light | 227 | 0 | 0 | 0 |  | 0.68 | 0.44 |  |  |  |  |  |
| Delivery pack | 227 | 0 | 0 | 0 |  | 0.95 | 0.95 |  |  |  |  |  |
| Suction device | 227 | 0 | 0 | 0 |  | 0.90 | 0.96 |  |  |  |  |  |
| Manual vacuum extractor | 227 | 0 | 0 | 0 |  | 0.21 | 0.57 |  |  |  |  |  |
| D&C kit | 227 | 0 | 0 | 0 |  | 0.00 | 0.39 |  |  |  |  |  |
| Newborn mask & bag | 227 | 0 | 0 | 0 |  | 0.94 | 0.98 |  |  |  |  |  |
| Bed or table | 227 | 0 | 0 | 0 |  | 0.82 | 1.00 |  |  |  |  |  |
| Blank partographs | 227 | 0 | 0 | 0 |  | 0.94 | 0.92 |  |  |  |  |  |
| Gloves | 227 | 0 | 0 | 0 |  | 0.96 | 0.99 |  |  |  |  |  |
| Domain: medicine and commodities |  |  |  |  |  |  |  |  |  |  |  |  |
| Tetracycline ointment | 227 | 0 | 0 | 0 |  | 0.74 | 0.92 |  |  |  |  |  |
| Injectable uterotonic | 227 | 0 | 0 | 0 |  | 0.90 | 0.97 |  |  |  |  |  |
| Injectable antibiotic | 227 | 0 | 0 | 0 |  | 0.59 | 0.75 |  |  |  |  |  |
| Injectable magnesium sulfate | 227 | 0 | 0 | 0 |  | 0.77 | 0.95 |  |  |  |  |  |
| Skin disinfectant | 227 | 0 | 0 | 0 |  | 0.88 | 0.58 |  |  |  |  |  |
| IV solution | 227 | 0 | 0 | 0 |  | 0.94 | 0.67 |  |  |  |  |  |

NA: Not applicable (i.e., item not asked in survey for this country)
