# Supplementary material for: Association between infrastructure and observed quality of care in 4 healthcare services: A cross-sectional study of 4,300 facilities in 8 countries
Source: PLoS Med. 2017 Dec 12;14(12):e1002464. doi: 10.1371/journal.pmed.1002464 (PMC5726617; doi:10.1371/journal.pmed.1002464)
Supplement: S2 Table — (DOCX) [file pmed.1002464.s003.docx]

S2 Table: Summary statistics of items composing country-specific clinical quality

|  | Items | Haiti | Kenya | Malawi | Namibia | Rwanda | Senegal | Tanzania | Uganda |
| --- | --- | --- | --- | --- | --- | --- | --- | --- | --- |
| Family planning | Ask age | 0.46 | 0.65 | 0.51 | 0.60 | 0.57 | 0.50 | 0.79 | 0.61 |
|  | Ask number of living children | 0.40 | 0.48 | 0.55 | 0.52 | 0.57 | 0.35 | 0.79 | 0.54 |
|  | Ask last delivery date | 0.30 | 0.40 | 0.33 | 0.21 | 0.53 | 0.41 | 0.56 | 0.48 |
|  | Ask last menstrual period | 0.48 | 0.44 | 0.35 | 0.28 | 0.48 | 0.65 | 0.57 | 0.32 |
|  | Ask reproductive intentions | 0.03 | 0.22 | 0.20 | 0.12 | 0.46 | 0.02 | 0.30 | 0.41 |
|  | Ask desired timing | 0.02 | 0.15 | 0.14 | 0.07 | 0.46 | 0.04 | 0.25 | 0.39 |
|  | Assess breastfeeding | 0.03 | 0.25 | 0.15 | 0.13 | 0.54 | 0.23 | 0.32 | 0.29 |
|  | Assess menstruation regularity | 0.14 | 0.39 | 0.22 | 0.54 | 0.61 | 0.42 | 0.42 | 0.55 |
|  | Assess smoking history | 0.02 | 0.11 | 0.02 | 0.03 | 0.33 | 0.19 | 0.02 | 0.11 |
|  | Ask STI symptoms | 0.08 | 0.17 | 0.09 | 0.16 | 0.32 | 0.08 | 0.13 | 0.37 |
|  | Ask chronic illness history | 0.05 | 0.21 | 0.08 | 0.13 | 0.39 | 0.25 | 0.28 | 0.35 |
|  | Check blood pressure | 0.71 | 0.82 | 0.37 | 0.92 | 0.87 | 0.92 | 0.38 | 0.68 |
|  | Measure weight | 0.51 | 0.82 | 0.56 | 0.83 | 0.88 | 0.87 | 0.41 | 0.60 |
|  | Prescribe FP method | 0.98 | 0.97 | 0.99 | 0.99 | 0.99 | 0.89 | 0.96 | 0.99 |
|  | Counsel on method | 0.81 | 0.92 | 0.84 | 0.92 | 0.98 | 0.70 | 0.84 | 0.96 |
|  | Discuss follow-up visit | 0.73 | 0.96 | 0.88 | 0.96 | 0.91 | 0.79 | 0.83 | 0.96 |
| Antenatal care | Ask danger sign | 0.69 | 0.77 | 0.60 | 0.85 | 0.88 | 0.66 | 0.66 | 0.74 |
|  | Ask pregnancy history | 0.47 | 0.79 | 0.79 | 0.90 | 0.83 | 0.75 | 0.67 | 0.88 |
|  | Ask start date of last menstruation | 0.59 | 0.94 | 0.83 | 0.96 | 0.95 | 0.84 | 0.79 | 0.96 |
|  | Measure weight | 0.82 | 0.97 | 0.76 | 0.92 | 1.00 | 0.95 | 0.85 | 0.85 |
|  | Palpate abdomen | 0.58 | 0.97 | 0.94 | 0.98 | 0.86 | 0.79 | 0.94 | 0.98 |
|  | Check oedema | 0.21 | 0.75 | 0.65 | 0.94 | 0.85 | 0.84 | 0.62 | NA |
|  | Conduct vaginal exam | 0.07 | 0.07 | 0.04 | 0.50 | 0.11 | 0.97 | 0.13 | 0.11 |
|  | Check blood pressure | 0.94 | 0.94 | 0.50 | 0.95 | 1.00 | 0.95 | 0.79 | 0.83 |
|  | Test for syphilis | 0.65 | 0.80 | 0.11 | 0.91 | 0.49 | 0.59 | 0.55 | 0.16 |
|  | Test for HIV | 0.40 | 0.92 | 0.70 | 0.83 | 0.72 | 0.73 | 0.80 | 0.65 |
|  | Test for anemia | 0.73 | 0.83 | 0.09 | 0.90 | 0.40 | 0.45 | 0.61 | 0.32 |
|  | Test blood group | 0.49 | 0.81 | 0.04 | NA | NA | 0.51 | 0.43 | NA |
|  | Test urine | 0.67 | 0.81 | 0.04 | 0.91 | 0.18 | 0.47 | 0.55 | 0.13 |
|  | Give tetanus toxoid injection | 0.24 | 0.81 | 0.44 | 0.53 | 0.68 | 0.68 | 0.75 | 0.79 |
|  | Prescribe iron or folic acid | 0.73 | 0.55 | 0.91 | 0.90 | 0.35 | 0.96 | 0.84 | 0.83 |
|  | Discuss nutrition | 0.47 | 0.50 | 0.42 | 0.70 | 0.41 | 0.32 | 0.38 | 0.40 |
|  | Counsel on use of ITN | 0.01 | 0.44 | 0.35 | 0.19 | 0.62 | 0.34 | 0.11 | 0.43 |
|  | Counsel on birth plan | 0.12 | 0.47 | 0.52 | 0.37 | 0.25 | 0.03 | 0.50 | 0.41 |
|  | Counsel on delivery supply | 0.05 | 0.18 | 0.53 | 0.23 | 0.22 | 0.09 | 0.57 | 0.54 |
|  | Conduct ultrasound | 0.22 | NA | 0.01 | NA | NA | 0.32 | 0.03 | NA |
|  | Write client card | 0.80 | 1.00 | 1.00 | 1.00 | 1.00 | 0.98 | 1.00 | 0.98 |
| Sick child care | Ask ability to drink | 0.21 | 0.46 | 0.28 | 0.44 | 0.25 | 0.08 | 0.30 | 0.66 |
|  | Ask normal feeding pattern | 0.42 | 0.50 | 0.19 | 0.58 | 0.44 | 0.46 | 0.40 | 0.68 |
|  | Ask feeding pattern in illness | 0.26 | 0.68 | 0.22 | 0.69 | 0.43 | 0.25 | 0.28 | 0.54 |
|  | Ask cough | 0.71 | 0.82 | 0.71 | 0.91 | 0.71 | 0.72 | 0.76 | 0.88 |
|  | Ask diarrhea | 0.45 | 0.46 | 0.41 | 0.59 | 0.37 | 0.55 | 0.61 | 0.67 |
|  | Ask fever | 0.75 | 0.88 | 0.80 | 0.89 | 0.70 | 0.81 | 0.93 | 0.92 |
|  | Ask vomit | 0.33 | 0.54 | 0.37 | 0.48 | 0.27 | 0.43 | 0.51 | 0.64 |
|  | Ask convulsion | 0.05 | 0.18 | 0.09 | 0.19 | 0.02 | 0.05 | 0.22 | 0.38 |
|  | Ask mother’s HIV status | 0.01 | 0.08 | 0.07 | NA | NA | 0.01 | 0.08 | NA |
|  | Ask ear pain | 0.05 | 0.12 | 0.06 | 0.24 | 0.06 | 0.06 | 0.17 | 0.17 |
|  | Count respiration | 0.19 | 0.24 | 0.17 | 0.44 | 0.14 | 0.19 | 0.14 | 0.18 |
|  | Measure weight | 0.78 | 0.47 | 0.20 | 0.88 | 0.64 | 0.34 | 0.15 | 0.51 |
|  | Plot weight | 0.13 | 0.21 | 0.05 | 0.48 | 0.08 | 0.09 | 0.04 | 0.15 |
|  | Measure temperature | 0.84 | 0.87 | 0.71 | 0.97 | 0.90 | 0.88 | 0.62 | 0.90 |
|  | Check mouth | 0.08 | 0.45 | 0.11 | 0.33 | NA | 0.21 | 0.16 | NA |
|  | Check pallor | 0.40 | 0.62 | 0.47 | 0.39 | 0.37 | 0.44 | 0.45 | 0.78 |
|  | Check oedema | 0.08 | 0.08 | 0.08 | 0.10 | 0.13 | 0.08 | 0.07 | 0.16 |
|  | Check vaccination status | 0.18 | 0.44 | 0.78 | 0.72 | 0.18 | 0.20 | 0.39 | 0.35 |
|  | Check vitamin A status | 0.03 | 0.20 | 0.02 | 0.31 | 0.18 | 0.09 | 0.04 | 0.16 |
|  | Check deworming status | 0.02 | 0.20 | 0.02 | NA | NA | 0.12 | 0.06 | NA |
|  | Explain medication | 0.59 | 0.63 | 0.53 | 0.95 | 0.48 | 0.50 | 0.38 | 0.45 |
|  | Recommend feeding during illness | 0.39 | 0.45 | 0.21 | 0.43 | 0.23 | 0.25 | 0.26 | 0.57 |
|  | Describe danger sign | 0.04 | 0.31 | 0.13 | 0.36 | 0.26 | 0.06 | 0.18 | 0.36 |
|  | Discuss follow-up visit | 0.27 | 0.56 | 0.24 | 0.57 | 0.03 | 0.56 | 0.23 | 0.50 |
|  | State diagnosis | 0.09 | 0.52 | 0.45 | 0.51 | 0.21 | 0.08 | 0.48 | 0.51 |
|  | Order HIV test |  | NA | 0.80 |  |  |  |  |  |
| Delivery care | Ask headache |  | NA | 0.23 |  |  |  |  |  |
|  | Ask bleeding |  | NA | 0.47 |  |  |  |  |  |
|  | Ask danger sign |  | 0.34 | NA |  |  |  |  |  |
|  | Check blood pressure |  | 0.73 | 0.63 |  |  |  |  |  |
|  | Check pulse |  | 0.60 | 0.48 |  |  |  |  |  |
|  | Wash hand |  | 0.28 | 0.51 |  |  |  |  |  |
|  | Wear gloves |  | 0.98 | 0.81 |  |  |  |  |  |
|  | Explain course of labor |  | 0.63 | 0.79 |  |  |  |  |  |
|  | Prepare uterotonic drug |  | 0.85 | 0.88 |  |  |  |  |  |
|  | Use partograph |  | 0.88 | 0.81 |  |  |  |  |  |
|  | Prepare newborn bag & mask |  | 0.41 | 0.34 |  |  |  |  |  |
|  | Administer uterotonic |  | 0.54 | 0.11 |  |  |  |  |  |
|  | Examine placenta |  | 0.58 | 0.79 |  |  |  |  |  |
|  | Examine for lacerations |  | 0.99 | 0.96 |  |  |  |  |  |
|  | Dry newborn |  | 0.55 | 0.99 |  |  |  |  |  |
|  | Place newborn skin to skin |  | 0.58 | 0.76 |  |  |  |  |  |
|  | Clamp cord |  | 0.38 | 0.62 |  |  |  |  |  |
|  | Check vital signs |  | 0.32 | 0.28 |  |  |  |  |  |
|  | Palpate uterus |  | 0.21 | 0.22 |  |  |  |  |  |
|  | Encourage immediate breastfeeding |  | 0.75 | 0.93 |  |  |  |  |  |
