# Supplementary material for: Association between infrastructure and observed quality of care in 4 healthcare services: A cross-sectional study of 4,300 facilities in 8 countries
Source: PLoS Med. 2017 Dec 12;14(12):e1002464. doi: 10.1371/journal.pmed.1002464 (PMC5726617; doi:10.1371/journal.pmed.1002464)
Supplement: S3 Table — (DOCX) [file pmed.1002464.s004.docx]

S3 Table: Correlation of infrastructure and observed clinical quality by service

A: Correlation of infrastructure by service in health facilities across 8 countries (N=4354)

|  | Family planning | Antenatal care | Sick child care |
| --- | --- | --- | --- |
| Antenatal care | 0.63 |  |  |
| Sick child care | 0.51 | 0.69 |  |
| Delivery | 0.41 | 0.55 | 0.58 |

B: Correlation of observed clinical quality in health facilities across 8 countries (N=4354)

|  | Family planning | Antenatal care | Sick child care |
| --- | --- | --- | --- |
| Antenatal care | 0.32 |  |  |
| Sick child care | 0.17 | 0.32 |  |
| Delivery | 0.27 | -0.06 | -0.10 |

C: Intra-class correlation of infrastructure and observed clinical quality in health facilities across 8 countries

|  | Infrastructure | Observed clinical quality | N countries | N facilities |
| --- | --- | --- | --- | --- |
| Family planning | 0.05 | 0.22 | 8 | 1842 |
| Antenatal care | 0.09 | 0.37 | 8 | 1407 |
| Sick child care | 0.08 | 0.38 | 8 | 4027 |
| Delivery | 0.00 | 0.03 | 2 | 227 |
