# Supplementary material for: Association between infrastructure and observed quality of care in 4 healthcare services: A cross-sectional study of 4,300 facilities in 8 countries
Source: PLoS Med. 2017 Dec 12;14(12):e1002464. doi: 10.1371/journal.pmed.1002464 (PMC5726617; doi:10.1371/journal.pmed.1002464)

S1 Fig: Assessment of potential non-linearity in associations between infrastructure and clinical quality in four health services


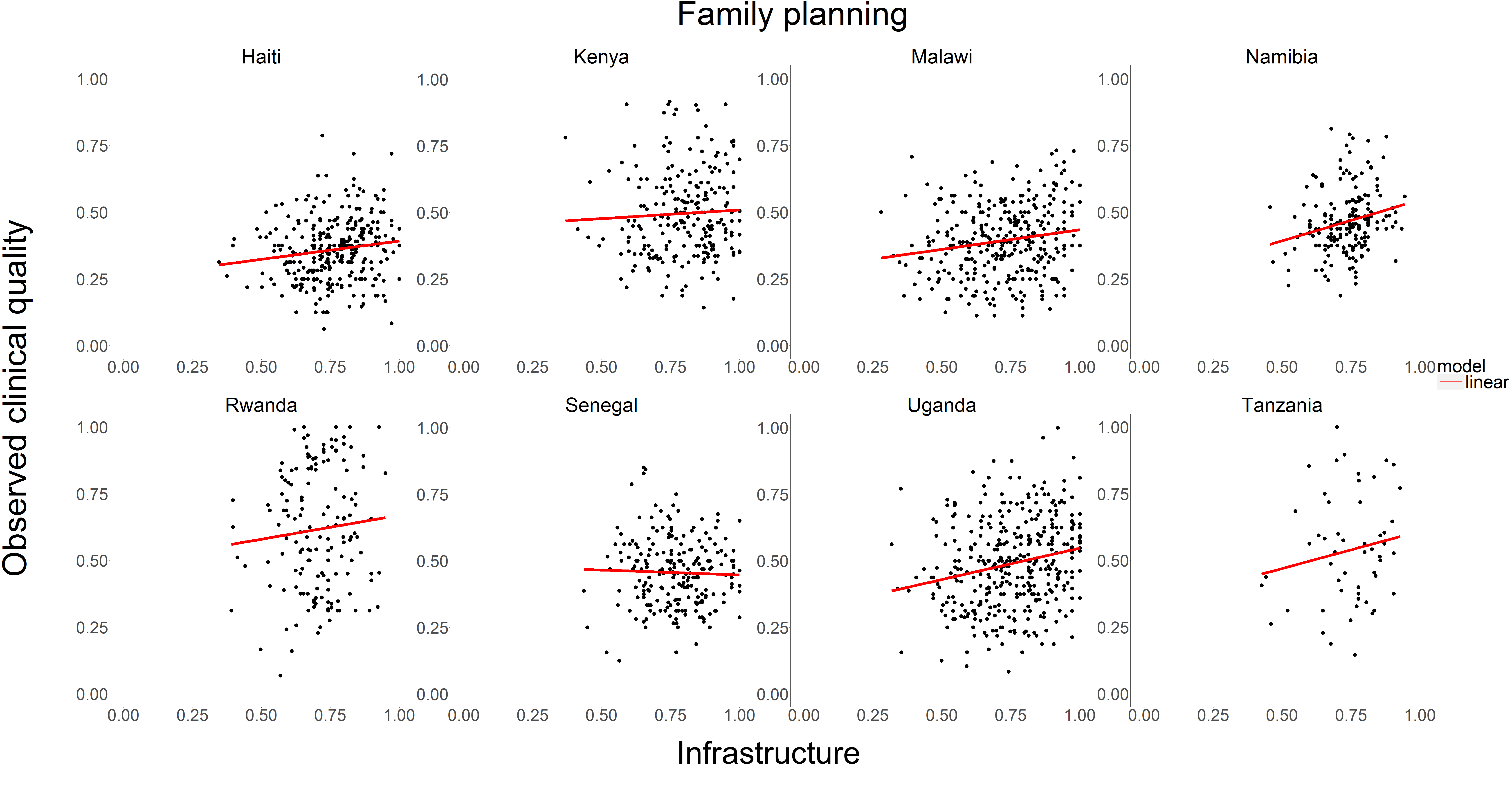


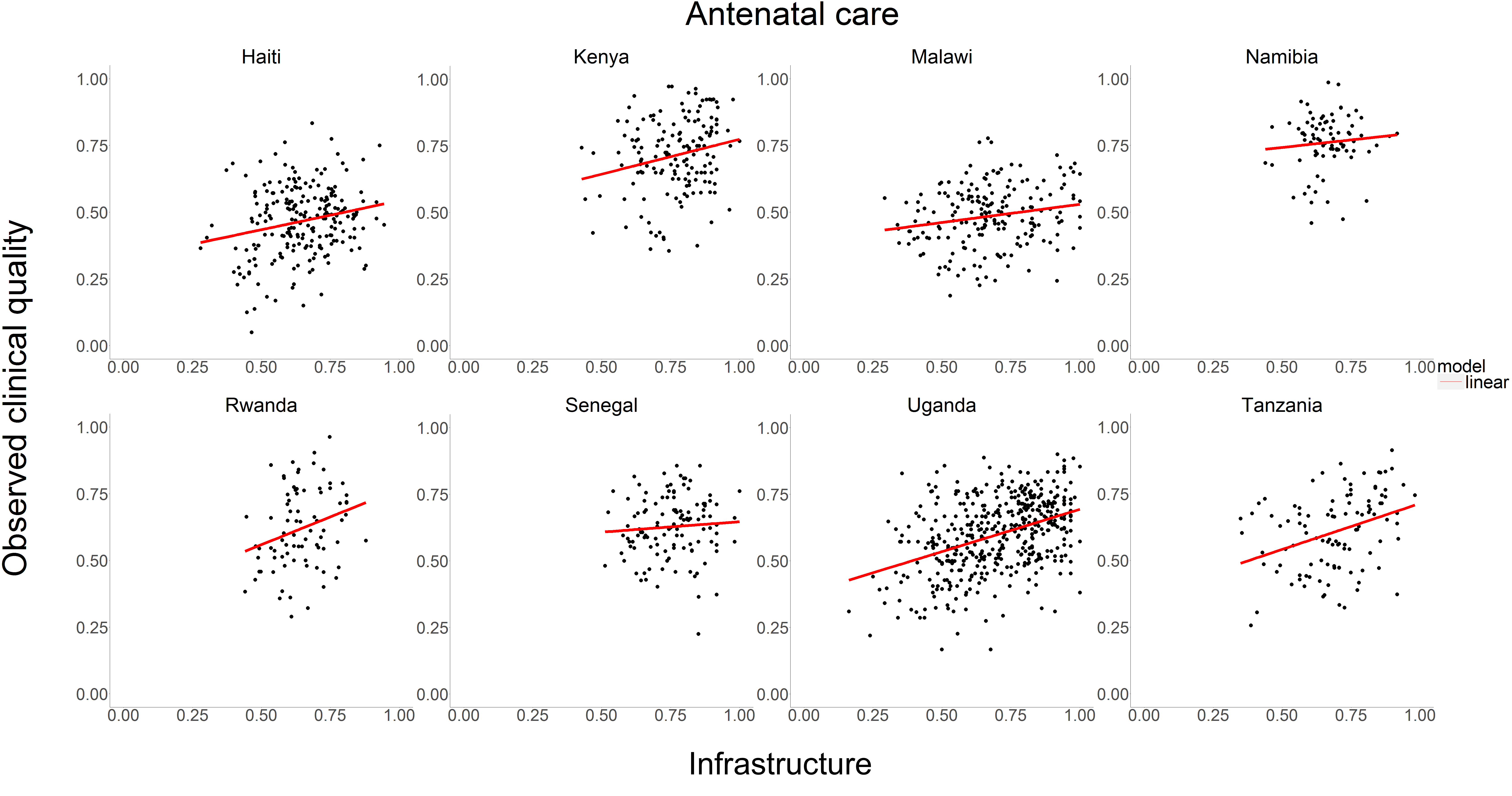


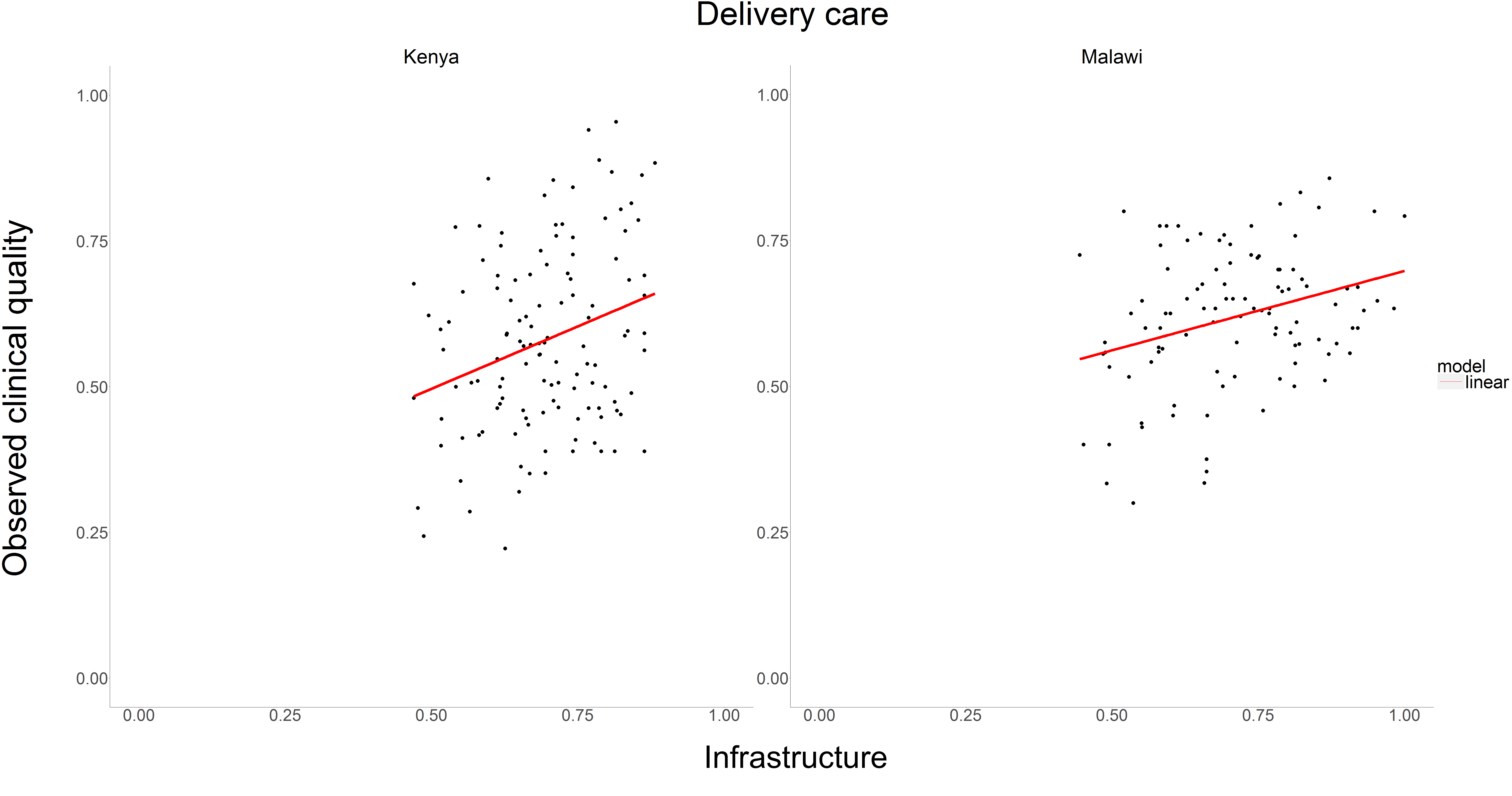


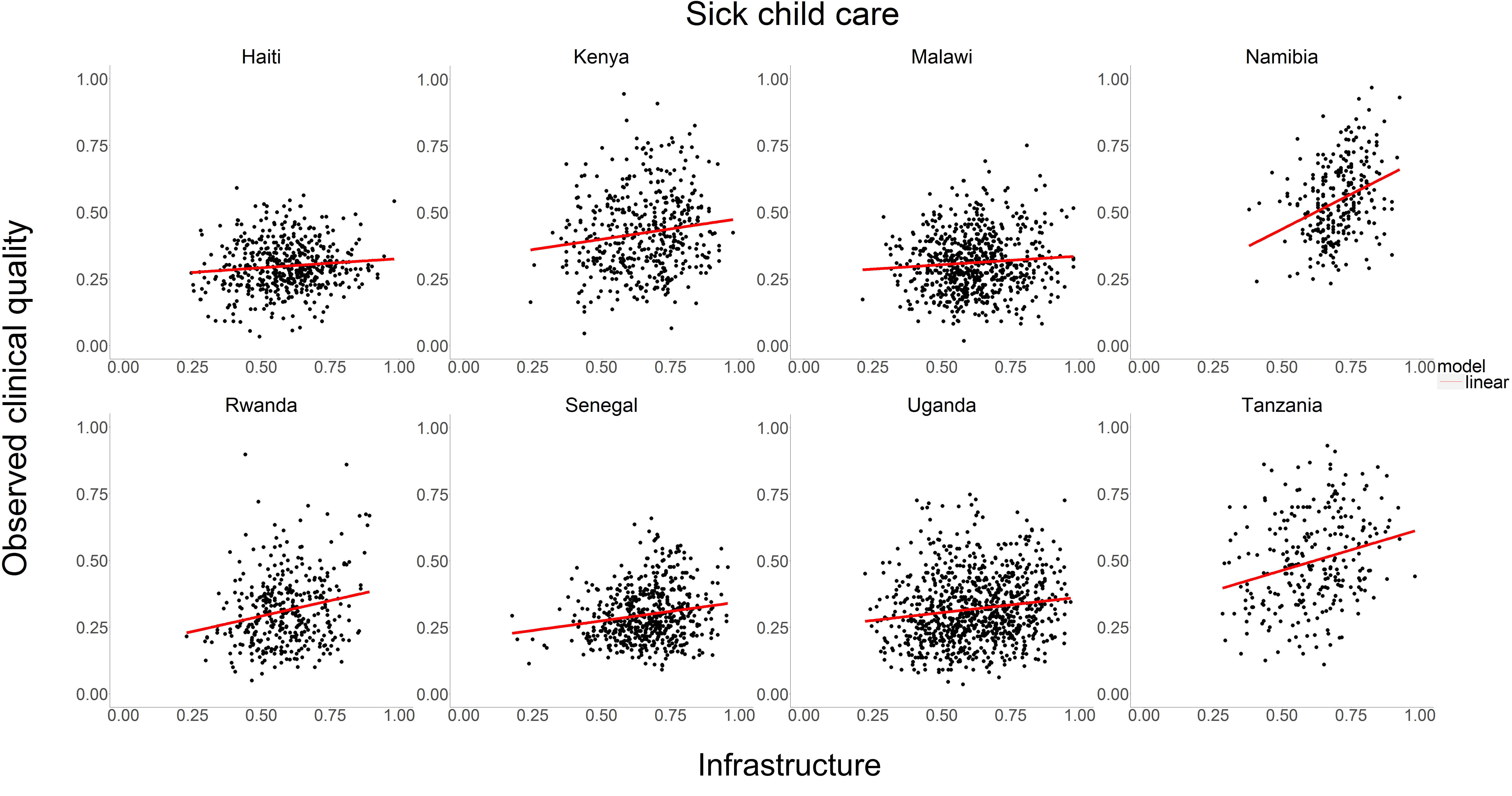

Supplement: S1 Fig — (DOCX) [file pmed.1002464.s005.docx]
